# Effect of lifestyle coaching versus care coordination versus treatment as usual in people with severe mental illness and overweight: Two-years follow-up of the randomized CHANGE trial

**DOI:** 10.1371/journal.pone.0185881

**Published:** 2017-10-06

**Authors:** Ane Storch Jakobsen, Helene Speyer, Hans Christian Brix Nørgaard, Mette Karlsen, Merete Birk, Carsten Hjorthøj, Ole Mors, Jesper Krogh, Christian Gluud, Charlotta Pisinger, Merete Nordentoft

**Affiliations:** 1 Mental Health Centre Copenhagen, Copenhagen University Hospital, Copenhagen, Denmark; 2 Institute of Clinical Medicine, Faculty of Health Sciences, University of Copenhagen, Copenhagen, Denmark; 3 Psychosis Research Unit, Aarhus University Hospital, Risskov, Denmark; 4 Department of Clinical Medicine, Aarhus University, Aarhus, Denmark; 5 The Lundbeck Foundation Initiative for Integrative Psychiatric Research, iPSYCH, Aarhus, Denmark; 6 Copenhagen Trial Unit, Centre for Clinical Intervention Research, Rigshospitalet, Copenhagen University Hospital, Copenhagen, Denmark; 7 Research Centre for Prevention and Health, Capitol Region, Copenhagen, Denmark; TNO, NETHERLANDS

## Abstract

The objective of this trial was to assess the long-term effect of the CHANGE lifestyle coaching intervention for 428 people with abdominal obesity and schizophrenia spectrum disorders on cardiovascular risk. In this randomized, superiority, multi-center clinical trial, participants were randomized to 12 months of either lifestyle coaching plus care coordination (N = 138), care coordination alone, (N = 142) or treatment as usual (N = 148). There was no effect after 12 months, but we hypothesized that there might have been a delayed treatment effect. Our primary outcome at two-year follow-up was 10-year risk of cardiovascular disease standardized to 60 years of age.

After two-years the mean 10-year cardiovascular-disease risk was 8.7% (95% confidence interval (CI) 7.6–9.9%) in the CHANGE group, 7.7% (95% CI 6.5–8.9%) in the care coordination group, and 8.9% (95% CI 6.9–9.2%) in the treatment as usual group (P = 0.24). Also, there were no intervention effects for any secondary or exploratory outcomes, including cardiorespiratory fitness, weight, physical activity, diet and smoking. No reported adverse events could be ascribed to the intervention. We conclude that there was neither any direct nor any long-term effect of individual lifestyle coaching or care coordination on cardiovascular risk factors in people with abdominal obesity and schizophrenia spectrum disorders. The trial was approved by the Ethics Committee of Capitol Region Copenhagen, Denmark (registration number: H-4-2012-051) and the Danish Data Protection Agency (registration number: 01689 RHP-2012-007). The trial was funded by the Mental Health Services of the Capital Region of Denmark, the Lundbeck Foundation, the Tryg Foundation, the Danish Ministry of Health, and the Dæhnfeldts Foundation.

## Introduction

Life expectancy in people with severe mental illness is reduced with up to 30 years compared to the background population[1], [2]. Age specific mortality rates for cardiovascular disease and other physical illnesses are 2 to 3 fold higher than in the general population with cardiovascular disease being the most common cause of death[1]. The increased morbidity and mortality can be explained by several risk factors, with factors related to lifestyle choices contributing largely[3]. Disparities in health care add to the excess mortality[4] as patients with severe mental illness are less likely to be tested for even the simplest of metabolic risk factors, such as blood glucose, dyslipidemia, and blood pressure[1]. Somatic health problems are therefore widely underdiagnosed and undertreated among people with severe mental illness[1].

Systematic reviews of randomised clinical trials assessing the effect of behavioral interventions for reduction of cardiovascular risk factors in people with severe mental illness, have shown small to moderate effects[5]. The current evidence is based on trials with short follow up—no longer than six months post intervention[6] and we lack knowledge about the long-term effects.

The objective of the CHANGE trial was to investigate the effect of an intensive lifestyle intervention that aimed to create sustainable enhancement of the cumulative health profile of people with schizophrenia spectrum disorders. The CHANGE trial used a comprehensive approach and addressed not only obesity, as the correlation between weight loss and mortality remains questionable[7]. Thus, we aimed at reducing several cardiovascular risk factors by

- Facilitating smoking cessation.
- Enhancing physical activity.
- Improving healthy dietary habits.
- Ensuring monitoring and treatment of already occurring health problems like hypertension, diabetes, and dyslipidemia.

The hypothesis was that a 12-months individually tailored lifestyle coaching intervention would reduce the estimated 10-year cardiovascular-disease risk of, and that the effects would be sustainable with positive results remaining at two-years follow-up. The trial design and the one-year results are described elsewhere[8], [9], and showed no effect of the intervention compared with treatment as usual on any outcomes. Based on our theoretical framework Motivational Interviewing and Stages of Change, participants might theoretically have moved from lower towards higher levels of motivation to change health behaviour, resulting in post-intervention weight loss, smoking cessation, improved cardiorespiratory fitness etc. The current report describes the two-years follow-up results in order to detect a possible late onset effect of the examined interventions.

## Methods

### Study design and participants

This study describes the two-years follow-up results of the CHANGE trial, which was a multi-center randomised clinical trial conducted in Denmark’s two largest cities (Copenhagen and Aarhus) during the period December 2012 through May 2014. The trial was investigator-initiated and independently funded. The trial protocol was published in 2015[8], see supporting informatin S1 File, and one-year post intervention results were published in 2016[9].

The participants were 18+ years old, and diagnosed according to the ICD-10 with schizophrenia (F20), schizoaffective disorder (F25), or persistent delusional disorder (F22)–confirmed at initial assessment by the Schedules for Clinical Assessment in Neuropsychiatry (SCAN)[10]–and with a waist circumference above the cut-off points for substantial risk of metabolic complications suggested by the WHO[11] (102 cm for men and 88 cm for women). Patients were excluded if they were currently pregnant or unable to give written informed consent.

The Copenhagen Trial Unit performed computerised central randomization with variable block sizes (9, 12, and 15) in a 1:1:1 ratio to one of three groups: lifestyle coaching, care coordination, and treatment as usual (CHANGE intervention); care coordination and treatment as usual; or treatment as usual alone. Randomisation was stratified according to sex, site, and risk of cardiovascular disease at baseline. The latter was split into high/low using cutoff points of15% risk of cardiovascular event for men and 7% for women in the next 10 [12], using the Copenhagen risk score[13] with age standardized to 60 years.

According to our protocol[8], all data published as main results of the study were blinded for statisticians and researchers. However, as the blinding was broken just prior to publication of the main results, the analyses performed in the present follow-up were not blinded.

### Interventions

#### CHANGE intervention

The CHANGE intervention consisted of one year of affiliation with a CHANGE coach, who was a healthcare professional (physiotherapists, occupational therapists, or dieticians) with clinical experience in psychiatry and with special training in smoking cessation, healthy dieting, and monitoring and treatment of lifestyle diseases. Lifestyle coaching was based on the transtheoretical model (stages of change)[14], motivational interviewing[15], and used an assertive approach[16]. The latter involved the coach offering at least one personal meeting per week or home visit of variable duration, often one hour, besides phone calls, test messages, and e-mails. The coaches aimed to motivate and support the participants in finding realistic and attractive options for daily-life physical activity, healthy dietary choices (involving purchasing of food and cooking sessions), and—where relevant—smoking cessation.

Each coach was assigned a maximum of 15 participants. All contacts with participants were registered by the lifestyle coaches. Besides the abovementioned intervention, the CHANGE intervention included care coordination and continued treatment as usual as described below. After 12 months of the CHANGE intervention all participants received treatment as usual.

#### Care coordination

Care coordination consisted of one year of being affiliated to a care coordinator who was a special trained psychiatric nurse, facilitating contact for the participants to the primary care sector in order to secure optimal treatment of physical health problems. Symptoms of cardiovascular disease, diabetes, or obstructive pulmonary disease were main focuses.

The care coordinator offered personal meetings, assistance at visits to the participants general practitioner, home visits, phone calls, and text-message contact with participant, and adjusted the frequency according to the individual need.

The care coordinator to participant ratio was 1:40 and as for the CHANGE intervention all contacts with participants were registered by the care coordinator. The care coordination included continued treatment as usual as described below. After 12 months of the care coordination intervention all participants received treatment as usual.

#### Treatment as usual

The participants randomised to treatment as usual received no extra lifestyle counselling or treatment of physical disorders besides what is offered from the public health care system. All people in Denmark have affiliation to a general practitioner with free consultation when needed. People with severe mental illness are treated in secondary mental health service, and people treated with antipsychotics receive at least yearly mandatory screening of metabolic risk factors. All patients retained contact with their usual general practitioner, who monitors and treats somatic diseases. All results from baseline and the two follow-up assessments were available to the participant or their usual carer, and the CHANGE research team contacted the participant or their usual carer if any result was of serious concern.

The interventions are described in greater detail and manuals are provided in the previous published trial protocol[8].

### Outcome assessments

Outcome assessments were done just after end of the interventions (described elsewhere[9]) and at two-years follow-up. The primary outcome was the estimated 10-year cardiovascular-disease risk, standardised to age 60 years using the Precard[13] score, a composite measure developed for two epidemiological studies inCopenhagen[12]. Precard consists of non-modifiable (sex, family history of cardiovascular disease, diabetes mellitus and prior heart disease) and modifiable factors (daily smoking (yes/no), total and high density lipoprotein (HDL) cholesterol, body mass index, and systolic blood pressure). The algorithm estimated the absolute risk of ischemic heart disease, myocardial infarction, stroke, or death within the next 10 years. We standardised this risk by estimating it as though all participants were 60 years of age, according to the recommendations by the European Guidelines on Cardiovascular Disease Prevention in Clinical Practice[17].

Our secondary outcomes were cardiorespiratory fitness (VO_2_max test performed on a bicycle), weight, body mass index, lung capacity (Easy-one^®^ spirometer), systolic blood pressure (average of three measurements from the right upper arm, with the participant sitting upright after resting for 10 minutes, and prior to the bicycle test), waist circumference, resting heart rate, HDL and total cholesterol, hemoglobin A1c, and self-reported level of moderate and vigorous physical activity (Physical Activity Scale[18]).

We also investigated the following exploratory outcomes: high sensitivity C-reactive protein, triglycerides, self-reported sedentary time, daily smoking (Fagerström Test for Nicotine Dependence)[19], diet (using a 24-hours recall interview and Food Frequency Questionaire[20]), symptoms of schizophrenia (cale for the Assessment of Positive Symptoms (SAPS)[21] and the Scale for the Assessment of Negative Symptoms (SANS)[22]), cognition (Brief Assessment of Cognition in Schizophrenia[23]), quality of life (Manchester Short Assessment of Quality of Life[24]), psychosocial functioning (Global Assessment of Functioning[25]), and perceived health[26].

### Statistical analysis

Power calculations are described in detail in the previous post intervention publication[9]. The original enrolment target was 450 participants, which provided 90% power to detect a difference between groups on risk of a cardiovascular event 2.5 percentage-points at 12 months.

Analyses were by the intention-to-treat principle. Linear mixed-model analysis with repeated measures and unstructured covariance matrix was used to calculate the estimated outcomes for all continuous measures. Analyses were adjusted for sex, center, and baseline cardiovascular-disease risk. Histograms were assessed for normal distribution, and any skewness was handled by the fact that the mixed-model analysis is robust for violation of the normality assumption.

For the one dichotomous outcome smoking status we used multiple imputation with the predictors: sex, age, randomisation, smoking status at baseline and at one year follow-up. 100 imputations and 20 iterations were conducted. Analyses were then performed using binary logistic regression on the imputed datasets, adjusting for sex, research center, and Precard.

Sensitivity analyses included complete-case analyses, outlier-removal (three standard deviations above or below estimated mean), and excluding participants without any contacts (per-protocol analysis).

We calculated post hoc the proportion of participants who gained or lost respectively 5 or 10 percent of their bodyweight during the two years using chi-square tests.

All statistical analyses were conducted using SPSS v. 22.

### Deviations from the protocol

Due to lack of referrals we only included 428 participants, i.e. 22 fewer than planned.

## Results

**Fig 1:**
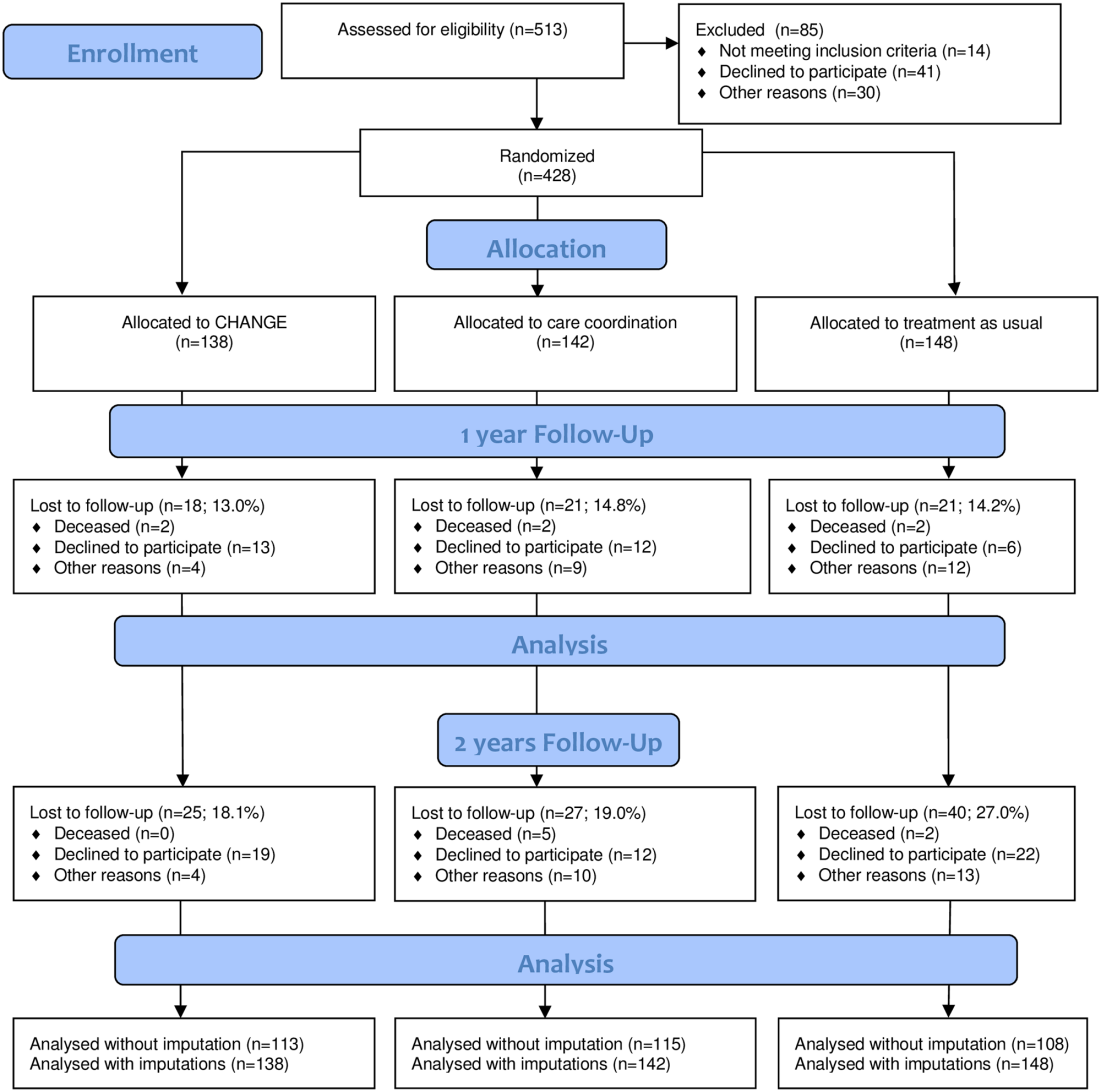
Flow diagram showing the process of recruiting and follow up through the CHANGE trial.

We included 428 participants from two sites (279 in Copenhagen and 149 in Aarhus). These were randomised to CHANGE intervention (N = 138), care coordination (N = 142), or treatment as usual (N = 148). The dataset is available, see supporting information S2 File, and the flow of patients through the trial can be seen in Fig 1. The mean age was 38.6 years (Range 18 to 68 years, Standard deviation (SD) 12.4), 240 (56%) were female. The majority had a diagnosis of schizophrenia (88%); while the remainder had either schizoaffective disorder (11%) or psychotic disorder not otherwise specified (1%). Most were prescribed antipsychotic medication (n = 408, 95%), 127 (30%) were on either olanzapine or clozapine, the two antipsychotic drugs with the highest risk of causing weight gain, diabetes and cardiovascular risk[27] and 166 (39%) received more than one antipsychotic agent. The mean duration of illness was 17.5 years (SD 1.9) with a range from 10 months to 64 years. The mean duration of untreated psychosis was 4.5 years (SD 6.7), with a range from 0 days to 36 years. The proportion of participants lost to follow-up was 21% at 2-years for the sample as a whole. There were no differences in the dropout proportions in the three groups (P = 0.39). Table 1 shows baseline metabolic, psychometric and medication characteristics for the dropouts and completers, respectively.

**Table 1:** Comparing baseline values for participants missing/not missing at two-years follow-up using ANOVA.

	Not missing		Missing		P
		SD		SD	
Sex (%Female)	56	56	51		0.370
Age (years, mean±SD)	39.04	12.36	36.99	12.47	0.157
Schizophrenia (%)	88		89		0.722
Employment yes/no (%)	3		3		0.929
Duration of Illness (years, mean±SD)	17.62	10.92	16.91	10.87	0.589
Supported housing (%)	09		15		0.093
Antipsychotics yes/no (%)	96.11		92.55		0.150
Polypharmacia yes/no (%)	39.82		35.11		0.409
Smoking yes/no (%)	50.60		57.45		0.241
Weight (kg, mean±SD)	102.86	21.26	103.30	23.84	0.863
BMI (kg/m2, mean±SD)	34.15	5.79	34.20	6.52	0.948
Waist circumference (cm, mean±SD)	114.55	14.60	114.74	15.61	0.911
Systolic blood pressure (mmHg, mean±SD)	127.78		126.85		0.577
Total cholesterol (mmol/l, mean±SD)	5.01	1.09	5.08	1.14	0.598
HDL (mmol/l, mean±SD)	1.22	0.37	1.24	0.42	0.659
Psychotic symptoms (SAPS, mean±SD)	2.12	1.61	2.45	1.55	0.074
Negative symptoms (SANS, mean±SD)	2.50	1.14	2.73	1.29	0.102
Cognition (BACS, mean±SD)	228.31	48.68	213.00	49.59	0.010
Level of functioning (GAF, mean ±SD)	44.03	44.03	42.63	7.93	0.121

SAPS—Scale for the Assessment of Positive Symptoms, SANS—Scale for the Assessment of Negative Symptoms, BACS—Brief Assessment of Cognition in Schizophrenia, GAF—Global Assessment of Functioning, HDL#x2014;high density lipoprotein·

The only difference between dropouts and completers was a slightly higher score in the completers for the cognitive test BACS.

Table 2 presents an overview of results on our primary and secondary outcome measures.

**Table 2:** Results for primary and secondary outcomes at 2 years using linear mixed-models.

	Change	Care coordinator	Treatment as usual	P (group x time)
**Primary outcome**				
10-year risk of cardiovascular disease (%)				
Mean±SD^a^	8.7±6.0	7.7±5.7	8.0±6.3	0.235
**Secondary outcomes**				
Weight (Kg)				
Mean±SD^a^	105.9±22.2	103.7±22.1	104.9±22.1	0.177
Body mass index				
Mean±SD^a^	35.6±8.6	34.4±8.7	34.4±8.6	0.131
Waist circumference (cm)				
Mean±SD^a^	114.8±17.0	114.9±17.1	117.0±16.8	0.834
Systolic blood pressure (mm Hg)				
Mean±SD^a^	129.1±13.0	130.1±13.5	128.3±13.4	0.522
Resting heart rate (beats/min)				
Mean±SD^a^	82.3±13.7	85.1±13.8	85.5±13.7	0.534
Cardiorespiratory fitness (ml O_2_/min/Kg)				
Mean±SD^a^	17.7±5.8	19.0±5.8	17.5±5.8	0.717
Forced expiratory volume (L/min)				
Mean±SD^a^	3.1±0.6	3.2±0.6	3.1±0.6	0.614
HbA1c (mmol/mol)				
Mean±SD^a^	39.7±7.8	39.3±8.1	39.3±8.0	0.731
Total cholesterol (mmol/l)				
Mean±SD^a^	5.02±1.0	4.94±1.0	5.04±1.1	0.693
HDL cholesterol (mmol/l)				
Mean±SD^a^	1.12±0.3	1.2±0.4	1.28±0.3	0.447
Triglycerides (mmol/l)				
Mean±SD^a^	2.1±1.7	2.1±1.8	2.3±1.8	0.665
Hs-CRP (mg/l)				
Mean±SD^a^	5.3±5.3	5.0±5.4	4.7±5.4	0.446
Moderate-vigorous physical activity (hours/week)				
Mean±SD^a^	2.6±5.9	2.9±5.9	2.8±6.0	0.811
Time spent Sedentary (hours/day)				
Mean±SD^a^	10.4±3.7	10.3±3.7	10.3±3.6	0.839
Daily smoking (yes/no)				
% (chi-square statistics)^b^	50.7 (0.03)	47.6 (0.01)	49.3 (0.01)	0.963
Intake of fruit (g/week)				
Mean±SD^a^	346.6±201.0	331.1±229.1	334.6±216.	0.392
Intake of vegetables (g/week)				
Mean±SD^a^	542.1±294.8	455.6±295.1	486.6±294.7	0.061
Intake of fish (g/week)				
Mean±SD^a^	119.0±113.9	137.0±114.0	139.7±113.7	0.746
Positive symptoms (SAPS global score)				
Mean±SD^a^	1.6±1.1	1.5±1.3	1.4±1.2	0.174
Negative symptoms (SANS global score)				
Mean±SD^a^	1.7±1.0	1.5±1.0	1.5±1.1	0.781
Cognition (BACS composite score)				
Mean±SD^a^	254.3±44.9	251.2±45.6	251.6±46.5	0.445
Quality of life (MANSA score)				
Mean±SD^a^	4.8±0.1	4.9±0.1	4.9±0.1	0.60
GAF total score				
Mean±SD^a^	48.6±10.8	48.1±10.7	48.1±10.78	0.792
Perceived health				
Mean±SD^a^	3.4±0.9	3.4±0.9	3.4±0.9	0.849

Hs-CRP—high sensitivity C-reactive protein, SAPS—Scale for the Assessment of Positive Symptoms, SANS—Scale for the Assessment of Negative Symptoms, BACS—Brief Assessment of Cognition in Schizophrenia, MANSA—Manchester Short Assessment of Quality of Life, GAF—Global Assessment of Functioning, HDL—high density lipoprotein· HbA1c—hemoglobin A1c

^a^ after mixed model analysis, adjusted for sex, research center and baseline risk of cardiovascular disease

^b^ after imputation with predictors: sex, age, randomization, smoking status at baseline and 1 year follow-up. 100 imputations, 20 iterations

There were no indications of statistically significant effects of any intervention compared to the other two on any outcome measure. We found that our primary outcome, mean age-standardised 10-year cardiovascular-disease risk was 8.7% (SD 6.0%) in the CHANGE group, 7.7% (SD 5.7%) in the care coordination group, and 8.0% (SD 6.3%) in the treatment as usual group (P = 0.24). In sensitivity analyses of complete cases, the 10-year risks were 9.0% (SD 5.9%) in the CHANGE group, 8.1% (SD 6.0%) in the care coordination group, and 7.8% (SD 6.1%) in the treatment as usual group (P = 0.08). Removal of six outliers or of three participants with no contacts had no statistically significant impact on the results.

Table 3 shows the percentages of participants meeting certain weight thresholds at two years after randomization, using chi-square tests.

**Table 3:** Percentages of participants meeting certain weight thresholds at two years after randomization using chi square tests.

	Change	Care coordinator	Treatment as usual	P-value
	% of participants			
Min 5% weight loss	25.36	19.72	16.89	0.200
Min 10% weight loss	11.59	11.27	8.78	0.697
Min 5% weight gain	20.29	20.42	21.62	0.954
Min 10% weight gain	12.32	6.34	8.78	0.219

The proportion of participants who at two years had lost at least 5% of their baseline weight was 25.4% in the CHANGE group, 19.7% in the care coordination group, and 16.9% in treatment as usual group (P = 0.20).

There were no statistically significant differences between groups for any of the other secondary or exploratory outcomes including cardiorespiratory fitness, BMI, blood lipids and hbA1c, smoking status and lung function, psychotic symptoms, subjective wellbeing, level of functioning and quality of life (Table 2).

ANOVA comparing mean weight and waist in participants receiving and not-receiving antipsychotic medication, respectively, did not show any significant difference, neither at baseline nor at two years follow-up. Also Pearsons corellations showed no association between antipsychotic medication dosage (as Daily Defined Doses) and weight or waist circumference, respectively, neither at baseline nor at two years follow-up.

### Adverse events

As seen in Table 3, the proportions of participants who gained 5% and 10% of their baseline weight, respectively in the three intervention groups were not statistically different. Thirteen participants died during the two-year follow-up period; two in the CHANGE group, seven in the care coordination group, and four in the treatment as usual group, respectively (P = 0.20). No deaths or other adverse events were reported that could be ascribed to the intervention.

## Discussion

### Principal findings

The aim of this randomized clinical trial was to investigate the long-term effects of a multi-domain personal coaching intervention, addressing the risk of cardiovascular disease in participants with schizophrenia spectrum disorders plus overweight in a community setting. The findings from 2-years follow-up of the intervention showed that neither the CHANGE intervention plus care coordination, nor care coordination groups achieved superior outcomes when compared with the treatment as usual group in reducing the cardiovascular-disease risk. No significant differences were found between the two intervention groups and the control group, neither in composite cardiovascular disease risk score, cardiovascular fitness, dietary habits, physical activity, psychiatric symptoms, cognition, perceived health or quality of life. These findings are in line with the previously reported results from the first one-year follow-up directly post-intervention, which also showed no significant difference between the three intervention groups in any outcome[9].

The lack of hypothesised intervention effect makes it irrelevant to talk of sustenance. However, one might have hypothesised a delayed treatment effect, which would be visible after a year post-intervention. This was, however, not the case, indicating that our motivational approach did not enhance motivation in the participants to change lifestyle choices, neither in the short- nor in the longer term.

### Strengths and limitations

CHANGE is to our knowledge the largest lifestyle intervention trial in people with schizophrenia spectrum disorder and overweight. External validity was high as we had very few exclusion criteria, an assertive recruitment process, and provision of an intervention without mandatory components. This methodological strength implies a challenge obtaining positive results, as recent research suggests that intrinsic motivation is important when it comes to adopting and maintaining of health promoting behaviours[28]. Using a less pragmatic design and including only trial participants who expressed readiness to change health behaviours may likely have led to more positive results, which, however, would only be applicable to the motivated parts of the population.

The trial used central randomisation, blinding of outcome assessors, data managers, and data analysts, and funding agencies had no impact on analyses or conclusions[8]. Our recruitment and retention rates were sufficient to not let power issues affect our conclusions. The intervention was manual-based and based on a solid foundation of evidence and theory[14], [15]. We compared lifestyle coaching with care coordination, which allowed us to tease out potential differential effects of lifestyle changes and those of improving monitoring and treatment of somatic illnesses and conditions. The intervention was sustainable, with a focus on identifying low-budget possibilities in the participants’ local environments.

Limitations include the fact that our primary outcome Precard has not been validated in a population with severe mental illness, and the generalisability of cardiovascular risk scores from the general population to people with severe mental illness has been questioned[29]. Another potential limitation regarding the Precard as outcome is that it included non-modifiable risk factors and therefore required a stronger effect on the modifiable factors in order to affect the composite measure. However, there was no indication of significant reductions in any of the separate modifiable risk factors, which makes this issue less relevant.

Another limitation to our trial is that we did not assess the participants’ level of motivation or stage of change at any time point, wherefore we do not have knowledge of possible enhancement of readiness to change, that might predict possible future positive results either by continuation of the CHANGE intervention or exploitation of this hypothesised increased readiness to change by a different kind of intervention.

### Strengths and weaknesses in relation to other studies

The results for our secondary outcomes; cardiorespiratory fitness and weight reduction are not in line with previous trials[5], which is probably attributable to different patient characteristics and types of interventions. Even though most previous evidence is reported to be favourable, the external validity is sparse and long-term trials are missing.

Another limitation consists of self-reporting of physical activity and eating behaviour. Potential recall bias may have led to random type II errors, and social desirability bias may have caused systematic errors. Direct measurements such as actigraphs may have been more accurate, but were not logistically and economically possible in our study. As for the measuring of dietary intake no method seems to be able to estimate habits and accurate intake without error. Assessing dietary habits under controlled or laboratory-like conditions is likely to influence dietary habits in the short-term, thus under-estimating regular eating patterns[30]. We believe that our chosen assessment methods were the best possible.

Regarding our theoretical framework for the intervention, the transtheoretical model and Motivational Interviewing are widely used in health promotion programmes for smoking cessation, healthy dieting, and physical activity[31], though documentation for the effect on lasting lifestyle change is sparse. Reviews of trials using the models in the general population found very low quality evidence for improvements in dietary and physical activity habits[32] or smoking cessation[33].

### Explanations and implications for clinicians and policymakers

Since only 42.8% of participants in the CHANGE group attended at least 50% of the planned sessions, it does not appear likely that increasing the number of sessions or decreasing the caseload would have yielded more positive results. Mean attendance was 24.6 sessions (SD 14.5, range 0–70). Making mandatory elements such as training groups, free healthy meals, or incentivising attendance or goals such as weight loss or smoking cessation with monetary or other kinds of reward may have led to positive results[34], but such interventions would be more expensive and doubtfully sustainable and thus would not easily be implemented in a real-world-setting.

An important notion is that even in the non-psychiatric background population, health behaviour changes are not easily achieved and maintained[35]. Behavioral interventions targeting both physical activity, diet and smoking cessation show modest yet significant benefits but evidence is generally sparse on long-term effects[36]. The landmark study Look Ahead found that intensive lifestyle intervention among patients with diabetes was successful in promoting weight loss and improving most cardiovascular risk factors as long as the intervention went on, but did not decrease cardiovascular events over an approximately 10-year follow-up period[37].

The control condition, treatment as usual, may partly explain the non-superiority of the care coordinator intervention as well as the CHANGE intervention, as health care is free and easily accessible in Denmark. Analyses of lipids or blood pressure at baseline did not indicate that participants in the CHANGE trial should change their medication. Furthermore, we only found two cases with no prior known history of diabetes in which HbA1c values indicated the presence of diabetes. Consequently, it cannot be excluded that an additional effect of care coordination over treatment as usual this trial could be present in countries with different health care systems. Also, merely being enrolled in the project, even in the control group, may have increased the awareness in participants on cardiovascular risk and the importance of healthy lifestyle choices (the so called Hawthorne effect), thus affecting their behaviour in a positive way causing a “spill over” effect with a hypothetical impact on the study outcome.

Our motivational approach based on the belief that it is possible to achieve behavioral changes even in unmotivated people may not be the right one to improve life for participants with schizophrenia. Suggestions for future research could be structural approaches—such as giving free healthy meals in institutions or small monetary rewards for healthy behavior such as loss of weight (within the limits of feasibility). Furthermore, multimodality-approaches could be considered, for example combining pharmacological treatment[38] (metformin, GLP-analogues, etc.) with behavioral intervention, or making use of internet- or telephone-based technologies. Several apps are available that target cardiovascular risk-factors for instance by exercise prescription or integration of patient information exchange with general practitioners, but the research field is still too young to provide evidence based solutions[39].

In conclusion, there were no indications of delayed or long-term benefits of the CHANGE life-style coaching intervention or care coordination over treatment as usual, in patients with abdominal obesity and schizophrenia spectrum disorders.

## Supporting information

S1 File Protocol for the CHANGE trial. (PDF)

S2 File Dataset including data from the CHANGE trial at baseline, 1- and 2 years follow-up. (SAV)
